# Economics of East Coast fever: a literature review

**DOI:** 10.3389/fvets.2023.1239110

**Published:** 2023-09-13

**Authors:** Aditi A. Surve, Jae Young Hwang, Shanthi Manian, Joshua Orungo Onono, Jonathan Yoder

**Affiliations:** ^1^School of Economic Sciences, Washington State University, Pullman, WA, United States; ^2^School for Global Health, Washington State University, Pullman, WA, United States; ^3^Department of Public Health Pharmacology and Toxicology, University of Nairobi, Nairobi, Kenya

**Keywords:** disease burden, cattle, costs, management, infectious disease, *Theileria parva*, ticks and TBDs

## Abstract

East Coast fever (ECF) is a cattle disease caused by a protozoan parasite called *Theileria parva* (*T. parva*). *Theileria parva* is transmitted among cattle by ticks. It is endemic in parts of central, eastern, and southern Africa and imposes an economic burden through illness and death of approximately a half of a billion U.S. dollars annually. This paper reviews existing science on the economics of ECF. We utilize a conceptual model that defines primary categories of economic costs due to ECF and use it to organize a synthesis of the literature on aggregate and micro level direct costs of the disease and the costs and benefits related to various ECF management strategies. We then identify knowledge gaps to motivate for future research.

## 1. Introduction

East Coast fever (ECF) is a cattle disease caused by a protozoan parasite called *Theileria parva* (*T. parva*). *T. parva* is transmitted among cattle by ticks. Once infected by ECF, an animal develops large lymph glands, becomes listless, stops feeding and coughs frequently. After the onset of these symptoms, the cattle usually suffers from fever followed by diarrhea and mucous discharges from the eyes and nose. The diseases can lead to morbidity-induced decline in cattle condition, milk production, draught capacity, and reproductive capacity, and death of the animal (1–4).

ECF imposes a substantial economic burden in several parts of sub-Saharan Africa (5) due to morbidity and mortality. The disease is common in central, eastern, and southern Africa and has been reported in 12 countries in the region: Burundi, Comoros, Democratic Republic of Congo (DRC), Kenya, Malawi, Mozambique, Rwanda, southern Sudan, Tanzania, Uganda, Zambia, and Zimbabwe (6), and kills at least 1 million cattle every year (7).

Management of ECF includes methods of prevention and treatment. Chemical acaricides, which are used to kill ticks for prevention of infection, have long been used in the form of dipping sprays, hand sprays, pour-ons and hand-dressing (5). However, acaricides are expensive, and ticks may develop resistance against them. Another prevention method is immunization through the Infection and Treatment method (ITM) developed by Radley et al. (8). ITM is a process where cattle are inoculated with a potentially lethal dose of cryo-preserved *T. parva* spores and simultaneously treated with a long-acting antibiotic (oxytertracycline). Di Giulio et al. (9) suggests that this immunization method maintains an attenuated infection that blocks more severe illness for up to 3 years but can be effective longer if infected ticks regularly re-infect immunized cattle. Antibiotics are generally used to treat non-immunized animals suffering from ECF. The first compound used in 1953 was tetracycline antibiotic. Due to their limited effects, other derivatives came up in the 1970's but are not used frequently because they are expensive (6).

This paper reviews existing science on the economic burden of ECF. We introduce a theoretical conceptual framework that distinguishes and defines the primary categories of economic costs due to the presence of ECF: Direct losses, prevention costs, and treatment costs. We then summarize the literature on aggregate and micro level direct costs resulting from cattle mortality and morbidity. We then review the literature on the costs and benefits related to various ECF management strategies based on empirical findings, including a review and discussion of cost-effectiveness and tradeoffs between the ECF management strategies. We then identify knowledge gaps in the science to provide a landscape for future research.

This review article contributes to the existing literature on ECF in several ways. First, this is the most comprehensive review of the existing literature on the economic burden of ECF to date. There are several reviews of ECF, but their coverage of economic dimensions of the disease is limited and tangential. Gachohi et al. (10) review how several factors such as agro- ecological zone, livestock production systems, environmental and socio-economic demographics influence the epidemiology of ECF. Di Giulio et al. (9) and Bishop et al. (11) review literature related to the biological and molecular developments for the methods to control the spread of ECF. While these reviews cover the biophysical dimension of ECF, they touch on the economic burden tangentially and in a relatively limited way.

Secondly, we provide a conceptual framework drawn from the economic literature on ECF and use it as a framework for organizing existing knowledge and analysis. We summarize the available estimates of economic burden at the household and more aggregate levels. We also examine the efficacy and economic tradeoffs between available prevention and treatment strategies.

Thirdly, from this synthesis we identify topical gaps in the literature on the economics of ECF, and discuss empirical methodological issues to the extent that it relates to confidence in the existing estimates and an understanding of the economics of ECF. These knowledge gaps can provide a useful road map for future research.

This paper is organized as follows. First, we comprehensively review the economic burden of ECF disease theoretically. Second, we analyze the direct economic costs for ECF from previous studies in the form of mortality and morbidity in cattle. Third, we look at the costs and benefits of control and management strategies. Last, we discuss implications based on our findings and explore directions for further research.

## 2. Methods

In this section we present a conceptual economic model that we then use to categorize, differentiate, and relate the various types of direct and indirect costs associated with infectious disease illness, avoidance, and treatment. We then introduce various economic tradeoffs in ECF management. We complete this section by describing the literature search methods supporting the review findings.

### 2.1. Conceptual framework

Adopting a model presented in Bennett et al. (12) [which itself builds on McInerney et al. (13)], the components of total cost associated with ECF infection and infection risk are described as,


(1) $$\begin{array}{l} C=\left(L\right)+\left(P+T\right),\;\text{where} \end{array}$$

- *C* = Total economic Cost of ECF.
- *L* = Economic Losses due to ECF illness.
- *P* = ECF Prevention costs.
- *T* = ECF Treatment cost.

Table 1 provides some examples of specific elements of each of these cost categories. Note that in all cases, monetization of losses and management activities is in principle possible, allowing for calculation of economic loss and cost metrics.

**Table 1:** Cost components with examples [adapted from Ikaal et al. (14)].

***L*: Loss due to illness**	**Mortality, abortion, milk production decline, premature culling, Condition and market value deterioration, dead animal disposal, and lost animal services (e.g., draught)**
*P*: Prevention cost	Acaricide chemical cost, labor cost for acaricide application and tick removal, ITM inoculation, and consultation fees
*T*: Treatment cost	Antibiotic treatment costs, and diagnosis and surveillance

Loss *L* in Equation 1 consists of the value of loss in expected output owing to the ECF disease, and in the case of ECF can include the potential benefits or value of cattle and its productivity that are eliminated or are otherwise not realized due to ECF. Building on Bennett et al. (12), an aggregate value of losses due to the disease can be estimated as,


(2) $$\begin{array}{l} L=p\times i^{d}\times \overset{J}{\underset{j=1}{\sum }}\left(i_{j}^{e}\times e_{j}\times v_{j}^{l}\right), \end{array}$$

where *p* is the size of the cattle population at risk, *i*^*d*^ is annual incidence of disease as a proportion of the population at risk. The summation accounts for a set of *J* identifiable and economically meaningful categories of disease effects, where $i_{j}^{e}$ is the incidence of distinct effect as a proportion of the infected population, *e*_*j*_ is the magnitude of physical disease effects and $v_{j}^{l}$ is the unit value of lost output or resource wastage (e.g., the net value price of milk lost). Losses *L* are often described as *direct* losses in that they follow directly and proximately from the consequences of illness. This differentiates from management costs that they incur to prevent and treat, and from the indirect effects of direct losses on households and the broader economy.

Prevention costs *P* can be summarized as,


(3) $$\begin{array}{l} P=p\times \overset{K}{\underset{k=1}{\sum }}\left(i_{k}^{p}\times v_{k}^{p}\right), \end{array}$$

where *p* is again the the cattle population at risk, $i_{k}^{p}$ is the proportion of population to which prevention measure *k* is applied, and $v_{k}^{p}$ is the unit cost of prevention measure *k* per animal (12). For example, chemical acaricides or hand-picking ticks off of animals are widely used to reduce the incidence of ECF (*i*^*d*^ and/or $i_{j}^{e}$ in Equation 3), and Infection and Treatment Methods (ITM) are often used to reduce the severity of ECF (*e*_*k*_ in Equation 3. Acaricide use reduces the exposure of ECF to cattle (15).

Treatment costs *T* include the cost of veterinary visits, medication costs, and extra labor costs attributable to ECF illness or risk of ECF illness. Aggregate treatment costs *T* in Equation 1 can be summarized as


(4) $$\begin{array}{l} T=p\times \overset{L}{\underset{l=1}{\sum }}\left(i_{l}^{t}\times v_{l}^{t}\right), \end{array}$$

where *p* is the number of livestock at risk (population at risk), $i_{l}^{t}$ is the proportion of the population to which treatment *t* is applied at cost of treatment per animal $v_{l}^{t}$ [building on Bennett et al. (12)]. The cost of a particular veterinary service depends on personal communication with a specialist. For example, Chi et al. (16) define the herd-level diagnostic costs to be the number of animals in a suspect herd multiplied by the cost per visit, whereas the medication cost is calculated by multiplying the number of infected animals times medication cost per case.

### 2.2. Economic tradeoffs in disease management

Herd owners use resources to reduce livestock illness-related production and asset losses by investing in prevention and treatment (13). Expenditure resulting from the disease includes increased management costs, disease treatment costs, and disease prevention costs (13). In terms of the model shown in Equation 2 through Equation 4, applying prevention inputs or activities and incurring their costs can reduce the incidence of the disease (*i*^*d*^), or can reduce *L* by reducing the incidence ($i_{j}^{e}$) or magnitude (*e*_*j*_) of one or more harmful disease effects. The costs of these prevention activities increase as the scope of prevention activities increase ($i_{k}^{p}$) and/or the intensity of the prevention activities increases (represented by $v_{k}^{p}$ subject to marginal input costs). Treatment inputs or activities can reduce losses *L* by reducing the magnitude of the effects of the illness (*e*_*j*_) given that an animal has become infected and ill, and perhaps avoid harmful effects completely given disease occurrence (thereby reducing $i_{j}^{e}$). Treatment costs increase with the scope of treatments ($i_{l}^{t}$) and the intensity of treatment ($v_{l}^{t}$).

McInerney et al. (13) discusses the productivity of treatment (*T*) and prevention (*P*) in terms of reducing losses (*L*). If one or both are highly effective at reducing diseases losses by reducing infection or consequence, total cost (*C*) can be reduced with expenditures on these inputs and activities. On one hand, a farmer could spend very little on treatment cost or prevention cost and bear high output losses *L*. At the other extreme, she could undertake all possible steps to reduce output losses to the minimum. If the productivity of *P* and *T* exhibits diminishing returns in the reduction of *L*, optimal investment in *T* and *P* likely exists between the above two extremes, and investment in prevention or treatment will stop when these investments provide less than one shilling of loss reduction. The perceived benefits of treatment and prevention by herd owners are affected by a great many things, including underlying disease risk, knowledge of the disease, herd characteristics, other herd management activities, the market price, availability, and time-costs of these inputs, as well as household resource constraints. This calculus at the household level provides a basis for discussing questions of the adoption and extent of use of treatment and prevention investments and activities.

The framework above can be used to identify, interpret, and understand the tradeoffs relating to the direct economic losses and management costs of infectious disease like ECF. However, there are indirect impacts on households and society related to broader income and substitution effects. At the household level, disease losses and investment in prevention and treatment reduce household income and wealth, which affect consumption opportunities. In terms of household production, disease losses and associated management costs reduce the rate of return on livestock and may alter households' decisions about their production asset portfolios. At the level of regional economies, disease may affect market prices and the flow of goods and services both regionally and internationally. The existing literature examines some of these indirect effects of ECF and we discuss these findings later in the article. In the remainder of this review, we use this conceptual framework to organize and categorize the economic burden of EFC losses and the economic dimensions of ECF prevention and treatment in relation to the existing literature.

### 2.3. Literature search and selection process

This paper originated as informal review of the literature on the economics of ECF to support a larger project on ECF management and treatment technology^1^. From this initial informal search, we shortlisted a set of papers published between 1989 and 2020 that are retrievable by a search on PubMed (http://www.ncbi.nlm.nih.gov/pubmed) and Google Scholar (https://scholar.google.com/) with the search phrase “ECF and economic estimate and livestock and cattle.”

After this initial informal review, we developed the conceptual framework presented in the paper as a way to organize the synthesis, and focused on studies that highlight and/or provide measurable direct and indirect economic consequences associated with ECF. This conceptual framework and an iterative process of identifying knowledge gaps provided a basis for additional literature search. A reviewer provided additional helpful suggestions. Although the process itself was iterative, a nearly complete list of articles reviewed in this article is retrievable by adding “theileriosis” to the search phrase provided above. Knowledge gaps identified in Section 7 were finalized after the full review of the relevant literature, and the estimates from the shortlisted studies were then reported in different categories of the economic burden of ECF losses corresponding to Equation 2. The remainder of this paper summarizes results from the literature identified by this search, organized according to the conceptual framework, and knowledge gaps are identified as a basis for future research.

## 3. ECF losses

As summarized in Equation 2 and its components, economic losses due to ECF are the product of a population at risk, infection incidence, and the consequences of infection, including morbidity and mortality and associated loss in livestock value that results. With this conceptual model as an organizational framework, we now review and synthesize the existing published information about these elements of disease costs in turn.

### 3.1. Population at risk

**Figure 1:**
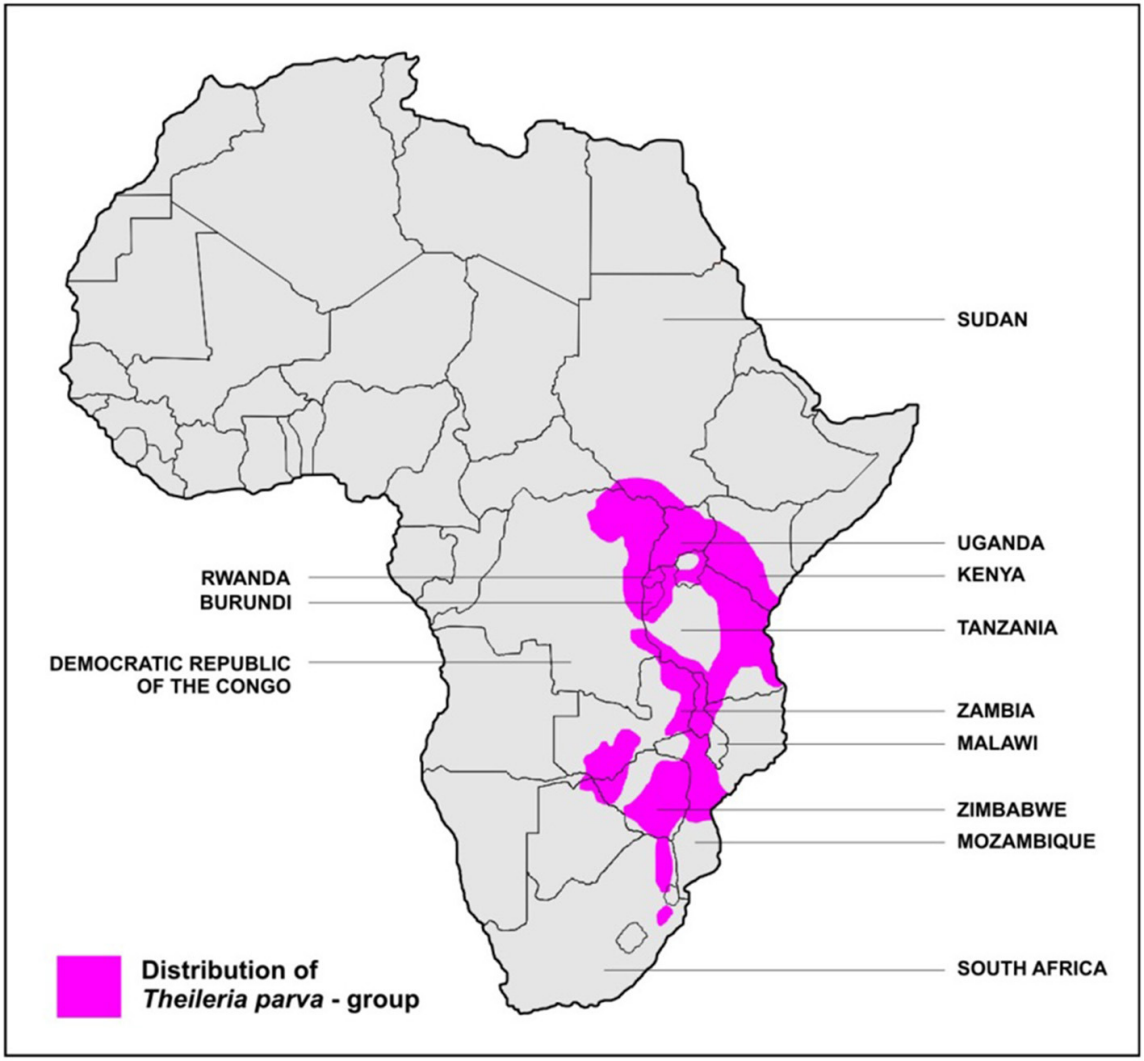
Stylized map of ECF distribution. Modeling by others [e.g., (20)] suggest a broader possible distribution. Source: Stoltsz (19). Available under Creative Commons attribution license.

*T. parva* is transmitted by the *Rhipicephalus appendiculatus* tick (17)^2^. ECF is common in central, eastern, and southern Africa and has been reported in 12 countries in these regions: Burundi, Comoros, Democratic Republic of Congo (DRC), Kenya, Malawi, Mozambique, Rwanda, southern Sudan, Tanzania, Uganda, Zambia, and Zimbabwe (19) (Figure 1). ECF kills at least 1 million cattle every year (7). de Villiers (20) identifies ECF as endemic in nine countries: Burundi, Kenya, Malawi, Mozambique, Rwanda, Tanzania, Uganda, Zambia, and Zimbabwe; and potentially viable but unreported in another eight countries (Angola, Botswana, Ethiopia, Lesotho, Namibia, Somalia, South Africa, and Swaziland). Gachohi (21) reports a similar mapping, and Olwoch et al. (7) find based on climate model projections that the Northern and Eastern Cape provinces of South Africa, Botswana, Malawi, Zambia, and eastern Democratic Republic of Congo show increases in ECF suitability.

### 3.2. Incidence and disease severity

Indigenous cattle have been found in some studies to be relatively resilient to ECF, even when raised under traditional extensive management practices in large areas where tick control is difficult and *T. parva* infection in cattle is common (10, 22). This epidemiological condition is referred to as “endemic stability,” where clinical disease and illness is rare despite high infection rates (23)^3^. This stability is believed to result from the cattle's high innate resistance, ability to quickly develop immunity, suitable ecological conditions for the parasite, and regular transmission, which boosts immunity in all age groups (4). Conversely, “endemic instability” occurs when fewer than 30% of the cattle become infected and immune, leading to a buildup of a susceptible population (*p* in Equation 2), and widespread clinical disease. This situation is usually found where animals are exposed to low levels of tick challenge. An ECF outbreak is likely to occur when susceptible animals are moved to a geographic area where ECF is endemic, or infected animals are moved to areas with endemic instability.

The severity of illness from ECF infection depends on several factors, including immunity from prior infection, parallel infection, type of ECF strain and dose, as well as the breed of cattle, among other factors which is *e*_*j*_ in Equation 2. ECF incidence can be higher when tick control measures are lax, resulting in cattle mortality and morbidity. In this section we present the direct production losses in Equation 2 bifurcated into two main categories of loss: mortality and morbidity, which are discussed next and summarized in Table 2.

**Table 2:** Loss: mortality, and morbidity analysis summary.

**Metric^*a*^**	**Rate(%)**	**Units**	**Source^*b*^**
**Mortality**			
R	8.5	Per ranch herds	[(15), UG]
R	8.2	Per pastoral herds	[(15), UG]
R	40	Per yearling zebu	[(24), KE]
R	19.4	Per animal	[(25), TZ]
AR	90	Per infected cattle	[(15), KE]
AL	28	Per infected cattle	[(12), KE]
**Morbidity**			
D	64	Per infected cattle	[(2), KE]
D	3	Per infected cattle	[(1), AF]
S	30	Per infected cattle	[(26), KE]
ML	25	Per infected cattle	[(1), AF]
MP	12.5	Per infected cattle	[(1), AF]
BL	5, 10	Per infected cattle	[(1), AF]

^*a*^R, Rate; AR, Abortion Rate; AL, Abortion Loss; D, Draft power; S, Slaughter value; ML, Milk loss; MP, Manure loss; BL, Beef loss.

^*b*^ISO Alpha-2 Code in citation indicates country of study (27).

### 3.3. Mortality and associated economic losses

Economic losses associated with mortality effects relate to loss in animal value (*p*, $i^{d}\times i_{j}^{e}$, and $v_{j}^{l}$ in Equation 2). While aggregate mortality estimates have not been published in the literature to our knowledge, several estimates of mortality rates have been published, as have the ECF burden via abortion effects. Mortality relates to illness severity parameter *e*_*j*_ in Equation 2.

Mortality rates due to ECF can vary depending on several factors such as the age and breed of the affected animal and the management practices employed. Ocaido et al. (15) conducted a longitudinal study in Uganda to observe the incidence of ticks and tick-borne diseases (TTBDs) on indigenous and cross bred cattle. The authors considered the incidence of Foot and Mouth Disease (FMD), Contagious Bovine Pleuro-Pneumonia (CBPP), ECF, and brucellosis, and found that ECF was the main cause of mortality among calves; of up to 8.5% of herds in ranch and 8.2% in pastoral herds. The mortality rate from ECF for untreated calves can be as high as 90% and from 10 to 30% in adult cattle. Spickler, (3) find that the mortality rate can be up to 100% in case of untreated taurine, zebu or sanga cattle prone to the disease. In the case of tropical theileriosis (caused by *T. annulata* rather than *T. parva*), the mortality rate for exotic or hybrid cattle breeds is estimated to be 40–90% but <5% for some indigenous animals (28). Kivaria et al. (25) found mortality to be 19.4% greater in the non-ITM animals compared to the ITM animals in Tanzania. Thumbi et al. (24) find that ECF was the cause of about 40% of deaths of shorthorn Zebu cattle in a sample from western Kenya.

East Coast fever has been identified as a cause of abortion in pregnant cattle (4). As per the findings, East Coast fever is a significant contributor to abortion in regions where the disease is prevalent. The study revealed that infected pregnant cattle, particularly in the early stages of pregnancy, can experience abortion rates as high as 90%. The disease-induced anemia and fever are typically responsible for fetal death and expulsion, leading to abortions. For abortion, Bennett et al. (12) analyzed the cost of cattle abortions as the reduction in milk yield and value of lost cattle associated with an abortion. They found that abortion causes a 28% loss in milk production and veterinary charges of £40. Sitawa et al. (29) reported an odds-ratio of 0.11 for abortions between vaccinated and non-vaccinated cattle. Thus the odds of ECF-induced abortion in non-vaccinated cattle are ~9 times higher than the odds of ECF-induced abortion in vaccinated cattle.

According to Kivaria (30), the cost of disposing of infected animals is a significant expense associated with outbreaks of ECF. In order to reduce the risk of contamination and the associated costs, farmers may need to implement appropriate biosecurity measures. If an animal is completely unused after an ECF death, then the cost of ECF mortality is the value of the animal at the time of death plus disposal costs. The disposal costs mainly include the cost of burying or incinerating the animal and disinfecting the area where the carcass was kept. If the carcass of an animal is used in some way, the economic loss associated with mortality is lower by the value of the carcass or its use.

### 3.4. Morbidity and associated economic losses

Economic consequences of ECF-related morbidity from ECF comes in various forms, including decreased milk production, loss of body weight, reduced cattle draft power, poorer cattle condition associated with lower market and slaughter value, and lower fertility rates. Based on longitudinal data from Uganda, Ocaido et al. (15) find ECF to be the main cause of morbidity among cattle, but they do not provide any estimates. Cattle that have recovered from ECF may continue to produce low milk output, suffer from reduced fertility, and experience delayed maturity, which imply long-term monetary losses (*v*_*j*_ in Equation 2) even when cattle survive.

A common clinical symptom of ECF in cattle is decreased milk yield. This reduction is linked to the disease's associated anemia and loss of appetite. Since milk production is a primary source of income for dairy farmers, the decreased milk yield caused by ECF can have significant economic consequences. Milk is also an important source of income for cattle owners who are not primarily dairy farmers, implying even larger economic consequences. Mukhebi et al. (1) reported a morbidity losses in surviving cows. An estimated milk loss of 25% was found in ECF infected cows.

Incidence of ECF in cattle can lead to loss of body weight (15), which may influence reproductive potential and longevity (31). It can also have an impact on beef production. A beef loss of 5% among recovered calves and 10% among pre-adult cows was reported (1). Optimal weight for cattle is instrumental to determine growth performance (32). Undernourished thin cows tend to be slower to re-breed at the time of calving as they lack nutrient reserves required for maximizing milk production (*v*_*j*_ in Equation 2).

ECF can cause a decrease in physical capacity and performance of cattle due to clinical signs and symptoms including fever, anemia, loss of appetite, and weakness. They can potentially provide less draft power which creates burden on livestock householders. A study conducted in Kenya reported that ECF reduced the work capacity of infected cattle by 28–64% compared to uninfected animals (2). Swai and Karimuribo (33) conducted a study in Tanzania and found that ECF-infected animals had a lower average weight-carrying capacity compared to non-infected animals. A reduction of 3% in draft capacity and 12.5% in manure production was estimated among clinically surviving ECF animals (1).

Another component of the direct loss calculation is reduced slaughter value ($v_{j}^{l}$ in Equation 2). Swollen lymph nodes are a common clinical sign in cattle infected with ECF. This condition may render the animal's carcass unsuitable for consumption, thereby leading to condemnation or downgrading at slaughterhouses. Chi et al. (16) suggest that the slaughter value of could be reduced due lower body weight because of the disease. Benedictus et al. (26) found that slaughter value of infected cattle was 30% lower than that of other cattle.

## 4. Management costs and benefits

In this section we look at different types of prevention and treatment measures used to control ECF among cattle. We also examine the costs and benefits associated with various techniques respectively based on empirical finding from literature. Finally, we review empirical papers from the literature to understand a cost-effectiveness and tradeoffs among different management measures of interest. Table 3 summarizes the results in the literature that are covered in more detail in the text.

**Table 3:** Prevention, and treatment analysis summary.

**Input^*a*^**	**Metric^*b*^**	**Value^*c*^**	**Units**	**Source^*d*^**
**Prevention**				
ITM	C	1.08	USD/animal	[(34), KE]
ITM	C	2.37	USD/animal/year	[(35), KE]
ITM	NPV	6,398	USD/farm/year	[(35), KE]
ITM	C	19.83	USD/animal	[(36), KE]
ITM	C	27.70	USD/animal/year	[(29), KE]
ITM	C	15.32	USD/animal/year	[(37), KE]
ITM	C	9.08	USD/calf/year	[(38), TZ]
ITM	NG	109	USD/farm/year	[(37), KE]
ITM	NG	925	USD/farm/year	[(29), KE]
ITM	NR	39.84	USD/farm/year	[(37), KE]
ITM	NR	6.81	USD/calf/year	[(39), KE]
ITM^*e*^	P	30.72	USD/calf/year	[(39), KE]
ITM	C	0.31	USD/farm/year	[(14), KE]
ITM	C	18.22	USD/animal/year	[(40), ZM]
ITM	E	0.033	Animal/year	[(41), KE]
ITM	OR	0.11	Animal/year	[(29), KE]
ITM	B	0.13	Litres/animal/year	[(41), KE]
ITM	OR	0.82	Animal/year	[(29), KE]
ITM	OR	1.08	Animal/year	[(29), KE]
ITM	L	6.08	USD/farm/year	[(14), KE]
Acar	C	2.99	USD/animal/year	[(35), KE]
Acar	C	309.61	USD/animal/year	[(25),TZ]
Acar	NPV	216.72	USD/animal/year	[(25),TZ]
Acar	BCR	1.7	Animal/year	[(25),TZ]
Acar	C	6.64	USD/animal/year	[(37), KE]
Acar	S	3.32	USD/animal/year	[(37), KE]
Acar	S	3.08	USD/animal/year	[(42), KE]
Acar	EX	2.83	USD/animal/year	[(42), KE]
Acar	C	16.17	USD/animal/year	[(40), ZM]
Acar	C	26.12	USD/animal/year	[(40), ZM]
Acar	C	40.44	USD/animal/year	[(40), ZM]
Acar	C	30.50	USD/farm/year	[(14), KE]
Acar	L	0.06	USD/farm/year	[(14), KE]
Acar&ITM	L	2.57	USD/farm/year	[(14), KE]
Acar&ITM	C	108.36	USD/animal/year	[(25), TZ]
Acar&ITM	NPV	751.63	USD/animal/year	[(25), TZ]
Acar&ITM	BCR	6.94	Animal/year	[(25), TZ]
**Treatment**				
ABX	C	45.65	USD/animal/illness	[(29), KE]
ABX	C	19.38	USD/animal/illness	[(38), TZ]
ABX	C	9.40	USD/animal/illness	[(41), KE]
ABX	C	10.77	USD/animal/illness	[(43), ZM]
ABX	C	17.34, 52.37	USD/animal/year	[(40), ZM]
ABX	C	7.66	USD/animal/illness	[(37), KE]
ABX&Acar	C	17.41	USD/farm/illness	[(14), KE]
ABX&ITM	C	0.29	USD/farm/illness	[(14), KE]

All monetary values from the literature reported in this table have been converted to 2023 US dollars (USD).

^*a*^ITM, Infect and Treat Method of vaccination; Acar, Acaricide; ABX, Antibiotics.

^*b*^B, Benefit; C, Cost; NPV, Net Present Value; NG, Net Gain; NR, Net Return; P, Profit.

S, Savings; EX, Expenditure; E, Elasticity; L, Loss; OR, Odds Ratio; BCR, Benefits Cost Ratio.

^*c*^All monetary values provided in the cited papers have been converted to USD Base 2023 (44, 45).

^*d*^Country of study is indicated by the ISO Alpha-2 Code in citation ISO (27).

^*e*^No treatment included (39).

The management tools popularly used against ECF in Africa include tick control, host (i.e., cattle) immunization and chemotherapy (*k* in Equation 3 and 4) (10). In some instances, an integrated combination of two or more measures can be used to control ECF. The tick control method mainly involves either manual tick removal or application of acaricides. Another highly effective prevention technique is the infection and treatment method of immunization. In terms of treatment chemotherapeutic drugs (i.e., antibiotics and antiparasite drugs) are widely used.

### 4.1. Treatment

Treatment relates to the reduction of disease effects $i_{j}^{e}$ once an animal is infected with a disease 2. Tetracycline antibiotics were widely used for treatment against ECF in the 1950's (6), but tetracycline is effective against ECF symptoms only during early stages of infection. Other compounds such as naphthoquinone came into use to treat ECF in the late 1970's. Animals infected by ECF can also be treated using antiparasitic drugs such as parvaquone and buparvaquone. More than one drug can be combined in cases of severe outbreak. These antiparasite drugs are effective in the initial stages of ECF illness but are relatively expensive ($v_{l}^{t}$ in Equation 4), and animals that are severely affected can die despite intensive care.

In estimating the direct costs of several related diseases in cattle, Chi et al. (16) assume that death and abortion losses were closely correlated with failure to receive veterinary treatment services when needed. Thus when the treatment cost *T* in Equation 4 is small or ineffective in reducing the effects of illness, the incidence of morbidity and/or mortality is high, which inflates total direct losses *L* in Equation 2.

Sitawa et al. (29) performed a cross sectional study based on a sample of 330 households and found that the estimated treatment costs were 2,200 KES (45. 65 USD). Homewood et al. (38) reported the average cost of treatment per cow in Tanzania was 19.38 USD. Ikaal et al. (14) analyzed treatment costs due to ECF when different prevention inputs such as acaricides and ITM were used. The treatment costs due to disease in case of acaricides only were 2,713.40 KES (17.41 USD) whereas due to ITM they were 45.70 KES (0.29 USD) per farm per year. Marsh et al. (41) estimated a treatment cost for ECF of 1,100 KES (9.40 USD) per animal. Billiouw et al. (43) carried out a study in Zambia to understand the burden of ECF epidemic that took place in the eastern region. The reported direct financial cost of the epidemic 10.77 USD per year per animal at risk. This estimate was based on loss of animal and cost of treatment only and was calculated over a period of 4 years.

### 4.2. Prevention

Application of acaricides is a traditional tick control method. The medium for application of acaricides can be in the form of dipping baths, hand sprays, pour-on liquids and hand-dressing. Exposure to ECF in endemic areas ($i_{j}^{e}$ in Equation 2) can be controlled by using acaricides (type of *k* in Equation 3) and implementing rotational grazing. However, this method is expensive (high $v_{k}^{p}$ in Equation 2) and enables ticks to develop resistance against it (increases *i*^*d*^ in Equation 3). It can be also harmful for the environment. In 1990, the annual estimated control costs related to acaricides in Kenya were USD 7.9 million (46). As of 2020, based on a cross sectional study, the annual cost of acaricide in Kenya was estimated to be 4,754.2 KES (30.50 USD) (14).

Hand-removal of ticks from cattle is a widespread method used by farmers to control tick populations and minimize the risk of transmission and infection of ECF and other tick-borne diseases in their herds. This method involves physically removing ticks from the animal's skin and is labor intensive. As a result, on-farm labor is the primary cost associated with this control method (47, 48).

The infection and treatment method (ITM) (a type of *k* in Equation 3), is an immunization method against ECF available in a form referred to as the Muguga Cocktail. The Muguga Cocktail is a live vaccine of ITM developed over several decades, with commercial production beginning in the 1990's (49). It has been the most commonly available ITM product for ECF. Perry (49) states that by 2016 over 1.5 million doses had been delivered, although its availability has been somewhat inconsistent (50). To date it has primarily been used in pastoral cattle production systems (51). It is composed of several tick species (Muguga, Kiambu 5, and Serengeti-transformed tick stocks) in order to increase the scope of effectiveness. The production method was introduced in the 1970's by the East Africa Veterinary Research Organization (EAVRO) laboratory at Muguga, Kenya. Alongside the administration of a vaccine, the animal is given an injection of an antibiotic, specifically 30% oxytetracycline. This antibiotic helps to restrict the impact of the parasite, thereby enabling the animal to develop an immune response without experiencing any substantial clinical effects (52). ITM causes a mild reaction (mild *e*_*j*_ in Equation 2) to the parasitic infection in the exposed animal, but the immunity that develops may be life-long. According to Babo Martins et al. (39), immunity lasts up to 3 years without any further tick infestations, or longer with continued exposure to infected ticks. Peters et al. (50) performed a systematic review of 61 research articles on the safety and efficacy of the Muguga Cocktail, and found “The majority of studies demonstrated or reported in favor of the Muguga Cocktail vaccine with regards to safety and efficacy.”

The benefits of ITM may come through reduced morbidity, mortality, and transmission. With early and effective treatment, the mortality rate can be reduced to <5% among calves and adult animals (4). When calves are vaccinated against ECF, they develop immunity to the disease, which may help to reduce the incidence of morbidity (10). Vaccination also reduces mortality rates and can improve the overall health and productivity of the herd. Sitawa et al. (29) found a mortality odds ratio of 2.1. In other words, non-vaccinated cattle have approximately twice the odds of dying from ECF compared to vaccinated cattle. According to Marsh et al. (41) in case of ITM, the elasticity of the death rate due to ECF was 0.033 implying that for every 100 calves that got vaccinated, 3.3 calves were saved from dying due to ECF.

To the extent that immunization reduces risk future of morbidity and mortality of an animal associated with the disease against which the animal is immunized, the immunization may be reflected in livestock markets. Babo Martins et al. (39) observed that selling price of an immunized cattle is 50% higher than a non-immunized one. Intuitively, a vaccinated animal is less likely to become ill and infect other animals, making it more valuable than a non-vaccinated animal.

The costs of ITM vaccination include the market cost of the vaccine and materials, labor costs for veterinarians and herd owners, and other possible costs. Mukhebi et al. (34) quantified the components of cost related to ECF immunizations. The estimated life-long immunization cost per animal was 40.36 KES (1.08 USD). This cost was calculated first by considering the difference between total and operating cost and then dividing it by total number of animals assumed to be immunized^4^. At aggregate level the, for a 30 year immunization plan starting from 1988, the estimated reported cost was 118.7 million KES (317 million USD) (34).

Two papers have analyzed adoption and willingness to pay for ITM. Randolph et al. (53) examine the willingness to pay for two vaccine products against ECF: ITM and a sub-unit vaccine product under development. They apply conjoint analysis and contingent valuation because market data were unavailable. Conjoint analysis was employed to evaluate the farmers preferences for vaccine attributes, and contingent valuation was employed to estimate the willingness to pay. Results suggest that smallholder farmers were willing to pay in the range of USD 25.19–USD 27.47 for ITM and between USD 25.97–USD 28.13 for the alternative sub-unit vaccine^5^.

Jumba et al. (54) examine ITM adoption rates, with a focus on differences in adoption between male and female heads-of-household. They find significant differences in adoption and willingness to pay between male and female heads-of-household, and that several factors affect adoption, but affect adoption differently between male and female household heads when analyses were performed separately for the two groups. Based on the pooled data they found that landholdings, household size, and group membership significantly affected the difference in adoption between the two types of house hold heads.

Nyangito et al. (35) ran simulations to compare how the use of ITM, acaricide and a combination of both compares in terms of net present value, benefit cost ratio and internal rate of return using a Technology Impact Evaluation System (TIES). TIES allows a ranking of alternatives based on stochastic dominance. Compared to the base alternative of using only acaricides, the authors found that the combination of ITM and 75% reduction in acaricide was most preferred. The costs related to a base case of spraying acaricides were reported to be 68.5 KES (2.99 USD). The estimated immunization cost was 54.4 KES (2.37 USD), whereas the net present value in case of ITM was found to be 146,000 KES (6397.73 USD) per farm per year. Moreover ITM strategies for ECF were economically more cost-effective than acaricides-based strategies ($v_{p}^{ITM}<v_{p}^{acaricide}$ in Equation 3). Minjauw et al. (55) also assessed the impact and financial implications of ITM in Zambia. According to the analysis, the break even price for was up to USD 25.9 per cattle ($v_{k}^{p}$ in Equation 3).

Kivaria et al. (25) conducted an analysis to assess the impact of immunization against ECF in Tanzania. They used a spreadsheet model to estimate the annual direct economic costs from milk loss, beef loss, acaricide application, and immunization and treatment services. Annual estimates were aggregated to obtain net present value and cost/benefit ratios under different tick control practices. Annual costs per animal per year were highest for acaricide application without immunization at 309.61 USD per animal per year, and lowest for immunization with a 100% reduction in acaricide application at USD 108.36 per animal per year. The net present value for immunization with a 100% reduction in acaricide application was highest at 751.63 USD per animal per year, and lowest for acaricide application without immunization at USD 216.72 per animal per year. The benefit and cost ratio for acaricide application without immunization and immunization with a 100% reduction in acaricide application were 1.7 (lowest B/C) and 8.42 (highest B/C), respectively (25) (Table 3). These results suggest that although immunization against ECF could support elimination of acaricide use for ECF alone, the authors note that some acaricide use would likely be necessary manage other tick borne diseases.

### 4.3. Tradeoffs among management strategies

In order to estimate change in profits due to adoption of vaccine to control ECF as opposed to acaricides, studies have used partial budget analysis. Partial budget analysis looks at changes in profits due to a particular change in input based on correlations as reflected in ANOVA comparisons between vaccinated and unvaccinated animals. The structure of partial budget analysis broadly consists of gains and losses incurred by the livestock owner due to changes one or more specific inputs (e.g., vaccinations, acaricide use, and labor). The gains further consist of the sum of extra revenue earned and extra cost saved, whereas the losses include sum of extra costs and revenue forgone. Thus, change in profit or the net change is obtained by taking the difference between gains and losses.

According to Sitawa et al. (29), households that vaccinated experienced a net gain of 44,575 KES (925.11 USD) whereas the non-vaccinating households had a net loss of 9,975 KES (207.02 USD) per cow per year. The result was mainly due to increased milk production and decreased ECF treatment costs for vaccinating households. They also reported that the estimated annual cost of ITM and antibiotic treatment was KES (27.70 USD) and KES (45.65 USD), respectively. However, the non-vaccinating households experienced 45% reduction in milk yield resulting in net loss. Similarly using partial budget analysis, Tenesi (37) found that the ITM technique helped livestock owners realize net gains of 4,261.45 KES (108.83 USD) per immunized calf. The estimated prevention cost of ITM was KES 600 (15.32 USD) and acaricide was 260 KES (6.64 USD) per cattle per year. The average cost of treating a calf up to 12 months of age was 300 KES (7.66 USD). ITM technique was also found to be profitable when sale of extra calves due to reduced mortality and expected price increase for immunized calves was not included.

D'haese et al. (40) conducted an economic costs analysis to study the burden of ECF in Zambia. It was done by computing the total output losses caused by ECF mortality and morbidity, tick damage, and the expenses incurred for treatment or preventive measures. The annual cost for acaricide application for plunge-dipping, hand spraying and pour-on was 16.17, 26.12, and 40.44 USD per animal per year, respectively. The cost for immunization per animal per animal was estimated to be 18.22 USD. The also reported a range of treatment costs between 17.43 and 52.37 USD per animal per year. The authors also found that compared to the base scenario of no intervention on average, immunization reduced the total economic costs by 90% whereas treatment reduced the cost by 60%.

According to Tenesi (37), the livestock owners realized a net return of 1,559.59 KES (39.84 USD) per immunized calf. Babo Martins et al. (39) found positive net gains using partial budget analysis indicating that controlling ECF through vaccination provides net returns compared to natural infection and treatment. They found an approximate net return of 7,250 TZS (6.81 USD) per vaccinated calf per year. Additionally, they calculated mean profit in case of no availability of medical treatment and all ECF cases leading to death. The resulting mean profit due to ITM in that case was 32,704 TZS (30.72 USD) per vaccinated calf. Sitawa et al. (29) reported an odds ratio of 1.08 for decrease in weight gain between vaccinating and non-vaccinated calves, which indicates that odds of losing weight are 1.08 times higher for unvaccinated animal than for a vaccinated animal, but this result is not statistically different from zero.

DeLay et al. (56) find that investment in prevention methods such as spraying of acaricide yearly on average increases milk yields by 11%. Marsh et al. (41) reported the milk productivity was 0.13 litres higher per adult animal with one additional vaccination. Ikaal et al. (14) used costs incurred by farmers due to reduced milk yield as a proxy for production loss due to ECF. The annual costs due to reduced milk yield for farms that used acaricides, ITM and combination of both were estimated to be 947.7 KES (6.08 USD), 9.10 KES (0.06 USD), and 401.20 KES (2.57 USD), respectively.

Studies have shown an increase in calving rates (*v*_*j*_ in Equation 2) from controlling tick-borne diseases (*P* and *T* in Equation 1). Increased calving rates due to prevention and treatment can lead to increased milk yields for households. According to Sitawa et al. (29) the odds of a decrease in calving rate and an increase in calving interval were lower for unvaccinated cattle than vaccinated cattle suffering from ECF. This is to say that vaccination would appear to negatively affect ECF infected cattle when it comes to calving rates and intervals, but neither of these differences were statistically different from zero.

A second potential benefit from ITM reducing acaricides is the opportunity to reduce acaricide use and associated costs. Further, reduction in acaricide use may allow for further infection of cattle with ECF, which is expected to boost immunity post-ITM (30). Lynen et al. (42) observed the changes in the average annual expenditure on acaricides per animal after adopting ITM. They observed that about 77% of farms from the sample reduced the frequency of acaricide application whereas 10% increased the same post immunization. Farms that reduced acaricide applications saved an average of 3,100 TZS (3.08 USD) per immunized animal, while farms that increased acaricide applications experienced an added expenditure of 2,847 TZS (2.83 USD) per immunized animal.

Tenesi (37) also observed that farmers who reduced the frequency of acaricide application post-immunization observed higher returns by saving more. Farms that reduced tick control frequency to once in two weeks experienced 50% reduction in acaricide expenditure. The tick control cost dropped from 260 KES to 130 KES per animal per year leading to savings of 130 KES (3.32 USD).

### 4.4. Prevention and treatment access and costs

The costs of ECF prevention and treatment are the sum of costs associated with several input goods and activities on-farm and in the input production supply chain. On-farm costs include time spent monitoring livestock health, administering prevention and treatment products such as acaricide and vaccinations, payment for veterinary services, transportation of livestock for care, and other activities. The market costs of purchased inputs are determined by production and distribution materials, activities, and their associated costs. In this section, we discuss the production costs, distribution challenges, and on-farm labor dimensions of the common ECF prevention and treatment methods including tick removal, acaricide use, ITM, and antibiotic treatment.

Hand-removal of ticks from cattle is sometimes used to control tick populations, and on-farm labor is the primary cost associated with this control method (47, 48). Farmers may need to hire additional labor to assist with tick removal or may have to spend more time themselves removing ticks, which can impinge on time spent on other farm tasks. Thus, the use of alternative tick control methods or improving tick removal efficiency can help reduce labor costs (57).

As previously discussed, acaricides are often used to control tick infestation on cattle. While less labor intensive than hand-removal of ticks, acaricides still require inputs in addition to the purchase price of acaricides, including training of operators, the purchase of equipment, and the disposal of used chemicals (30, 48). Furthermore, the repeated use of acaricides can lead to the development of resistance in ticks, reducing the efficacy of the chemical over time. However, acaricides are widlely available and stable, relatively low-cost, and are commonly used for prevention of tick-borne diseases.

Various treatments exist for cattle ill from ECF. The supply chain for ECF treatment can be divided into several stages, including production, distribution, retail, administration and monitoring. The specifics of the supply chain for ECF treatments can differ based on the region and market. Farmers in certain areas may encounter challenges in obtaining the necessary treatment for their animals due to limited access to veterinary clinics or distribution channels (16). In addition, there is a possibility of relapse after treatment, and animals may require additional rounds or alternative drugs if they exhibit signs of ECF in the future, thus increasing future costs (58).

ECF ITM vaccines have been produced and distributed since 1970's. The first vaccine was developed by scientists at the East African Veterinary Research Organization (EAVRO) in Kenya, using a weakened form of the *Theileria parva* parasite. However, there are several challenges when it comes to development and distribution of ECF vaccines in African countries imposing additional costs. Firstly, ITM distribution requires a cold chain, which can be expensive. The components of cold chain require a coordinated and consistent sequence of events, including having a temperature-controlled infrastructure to store and transport vaccine (liquid nitrogen is often used for this purpose), skilled management to supervise, and timely transport of the products. This leads to an additional cost to obtain the vaccine, which can be as high as USD 10 per cattle. Secondly, the Muguga Cocktail vaccine historically has been distributed in doses of 40 per package, which often exceeds the preferred number of doses for individual livestock owners, especially owners of small herds. The quantity of the vaccine package drives up the cost making it expensive for smallholder farmers to purchase, or calls for coordination activities to coordinate delivery of a full set of doses across multiple herds in a timely manner before vaccine spoilage. Thirdly, poor transportation infrastructure and distances often lead to logistical complications and high costs for ECF vaccine delivery in much of rural Africa where ECF is endemic. In previous studies, human vaccine delivery is found to be better developed than livestock vaccine delivery. For example, Schelling et al. (59) suggest that livestock vaccine delivery in Southern Sudan depends on human vaccine delivery infrastructure.

A number of articles discuss the political-economic complexity of vaccine markets as both a symptom of private market ineffectiveness for vaccine delivery and a cause of ineffective at supporting private and/or optimal social demand for various reasons. Angelmar and Morgon (60) suggest that private demand is weak relative to socially optimal demand because of positive externalities associated with vaccination (61–63). The existence of positive externalities provide an economic motivation for public involvement in the supply and distribution of vaccines. However, such direct public-sector involvement adds complexity as well that may reduce the efficacy of delivery along other dimensions of healthcare supply and ultimately reduce uptake. Schelling et al. (59) make the point that there are opportunities to coordinate human health delivery systems with livestock health delivery systems to improve livestock healthcare delivery, but few examples of such coordination exist to date. While there is an extensive literature on the economic foundations and effects of public policy on vaccine supply and demand, little of it has focused on ECF specifically.

## 5. Total and aggregate economic costs of ECF

In the sections above we describe the findings of previous studies relating to direct losses and management costs. We now discuss estimates that synthesize this information in terms of total economic costs at the micro-level, and then review the estimates of aggregate (macro-level) economic costs at regional, national, and international scales.

### 5.1. Total economic costs

Total economic cost *C* is the sum of losses *L* and prevention and treatment costs *P* and *T*. Ikaal et al. (14) estimated the economic losses due to ECF by summing the value of reduced milk yield to represent *L*, and the costs of acaricide use and vaccination. The estimated economic loss (C from 1) incurred by farms using only acaricides for prevention was highest at 111.94 USD per animal per year. Economic losses (C from 1) from using both acaricides and vaccination was estimated to be 62.44 USD and 2.13 USD when only vaccination (not acaricide) was pursued. Thus, the total economic loss (C from 1) for farms using only acaricides was 1.8 times higher than the combination of acaricide and vaccines, and 48.8 times higher than vaccine only. Sitawa et al. (29) also conducted similar analysis using the different cost elements to obtain the total economic costs. The total mean economic cost of ECF per cow per year was found to be 34,875 KES (281.66 USD). As seen in former study Ikaal et al. (14), the disease prevention costs were higher than treatment costs.

### 5.2. Aggregate costs

Mukhebi et al. (1) estimated the aggregate losses from ECF and benefits of ECF prevention and treatment for eleven countries across Africa. They obtained or derived country-specific parameters from assumptions, expert opinions in the field of ECF disease burden and literature based on data of 1989. According to the results, the total annual economic direct losses due to ECF were estimated for eastern and southern Africa to be USD 168 million, or 414 million in USD 2023 (*L* in Equation 1). The authors presented ranges of parameters that were used in calculation of losses in eleven affected countries^6^. The losses comprise two main categories: beef loss and milk loss ($v_{j}^{l}$ in Equation 2). The two categories further included mortality and morbidity losses. Additional losses were included for categories such as manure loss, acaricide application, research costs, animal traction. Milk loss was the largest contributing about 47% of total, followed by acaricides, loss of traction, and beef.

McLeod (46) report estimates of aggregate production losses related to several tick borne diseases, including ECF. This calculation takes into account the incidence of diseases within each system and the resulting production losses. The overall losses are at a national level determined by aggregating the results of each system and taking into account the prevalence of each system and the size of the national herd. The annual mortality production losses due to ECF in Kenya were estimated to be USD 69.7 million. From 1980 to 1990, milk production in Kenya was reported to grow from ~2 million to 2.4 million metric tonnes annually. Morbidity losses in terms of milk loss and liveweight were reported to be USD 15.5 million and USD 2.0 million.

## 6. Indirect and broader economic impacts

The economic costs of ECF have indirect economic consequences in the household, community, and region. ECF reduces in-kind and financial income for livestock-keeping households due to its impact on cattle productivity and value. In addition, household resources spent on ECF prevention and treatment would be available for household consumption or other production investments and activities were it not for the ECF burden. The literature includes analyses of indirect effects of ECF on food consumption and nutrition, child health, and schooling.

The effect of ECF on milk production and its consequences for the household are a case in point. Behnke and Muthami (64) estimate that milk accounts for about 70% of the gross value of livestock in Kenya, and Delgado et al. (65) estimate that milk contributes 60% of the total rural household income in sub-Saharan Africa. Such dairy production can enhance human welfare and reduce poverty. Improved milk yields can also translate into indirect intra-household benefits, such as higher educational attainment and improved nutritional outcomes (41, 56, 66).

Mosites et al. (67) also argue that cow milk consumption can improve early childhood growth. Average livestock numbers owned over the previous 9 months can positively affect child growth rate when other factors are constant. They emphasized that household livestock production might benefit child nutrition, health care, education, and sanitation. Additionally, direct consumption of meat, eggs, or dairy products can promote children's dietary diversity. According to the Kenya Dairy Board (2008), the average productivity per cow is estimated to be 5–7 litres per day. However, most householders are resource-poor or are vulnerable to environmental changes.

Marsh et al. (41) examined decision-making to adopt vaccination against ECF and found that vaccination yields considerable net income advantages. This is primarily due to decreased livestock mortality, amplified milk production, and reduced need for antibiotic and acaricide treatments. The households utilized the additional income obtained through vaccination against East Coast fever to fund childhood education and food expenses. They found that a 10% increase in ECF vaccination causes a 0.88% increase in education expenditure and a 0.56% increase in food expenditure.

Mwilla et al. (68) conducted an analysis in Zambia using Productivity Adjusted Life Years (PALYs). The objective was to estimate the non-monetary societal burden of ECF. The primary reasons for cattle keeping in Zambia include draught power, showcasing social prestige, fulfilling dowry obligations, providing transportation services, generating income through sales during financial bottlenecks, producing milk, serving as a source of employment, and occasionally using them for meat production as a result of which ECF can lead to substantial social and economic distress for the farmers. PALYs was calculated by summing years of life lost due to premature mortality (YLL) in the cattle population and the equivalent healthy years lost due to disability (YLD). As per estimates a loss of 517,165.40 PALYs was reported due to ECF.

## 7. Research and knowledge gaps

We discuss several knowledge gaps on topics relevant to the economics of ECF, and identify research opportunities worthy of consideration: These fall into knowledge and research gaps relating to morbidity, mortality, management cost estimation methods, data needs and limitations, scope of ECF impact, the role of institutions for ECF management, ITM supply and demand estimation, indirect impacts of ECF, the economics of on-farm labor, and the intersection of ECF and trade. Our discussion is focused specifically on the economics of ECF, but it overlaps in some general ways with Perry and Randolph (22), who consider economic impact assessment of parasitic diseases more broadly.

### 7.1. Morbidity

While there have been some studies on the economic impact of ECF, there is still a knowledge gap in understanding the scope of morbidity costs associated with ECF. Studies to date have mainly focused on estimating the direct costs associated with ECF such as loss of milk production, loss of weight, loss of draught power, decrease in calving intervals (2, 14–16, 26, 33, 41). Nonetheless, there is little information about the long-term effects of ECF. Further, existing findings related to morbidity costs are not homogeneous across studies, which makes it difficult to make comparisons. Morbidity costs have been defined and measured differently, which can lead to disparate estimates that are hard to interpret. With existing research in mind as context, additional research can shed additional light on the sources of variation in morbidity and mortality and the economic costs associated with the ECF burden.

### 7.2. Mortality

In terms of ECF-related mortality, several studies have looked at death rates and abortions (4, 24, 29, 69, 70). However there is not much information or empirical research on estimates of costs related to disposal of ECF infected animals. Costa and Akdeniz, (71) discuss the importance on further research needed on the disposal of infected animals to prevent the spread of tick-borne diseases. This is an important component of cost related to ECF as it requires farmers to undertake appropriate bio security measures to minimize the risk of contamination and the associated costs.

### 7.3. Methods of management cost estimation

A number of studies in the literature have used partial budget analysis to estimate the economic costs of ECF (29, 37, 39). Partial budget analysis is a tool popularly used in farm management to measure the changes in profits because of a potential change. However, there are some drawbacks of using this method. Firstly, the final estimate is measured after evaluating two alternatives. The cost and returns only adhering to a partial change are considered, whereas changes in other costs and returns are not accounted for. Secondly, it does not discount for the time value of money. Hence estimates do not account for the tradeoff between the value of money today vs. tomorrow. Thirdly, partial budget estimates are not a full measure of profitability, but an estimate of an alternative to current operations. As such, these estimates may not capture important substitution behaviors in response to ECF, and therefore may be biased in one direction or another, limiting the validity of estimates at the household and aggregate level of the full economic burden of ECF.

### 7.4. Data for cost estimation

Data collection is costly, and many studies therefore are based on small sample sizes. As a consequence, estimates of burden and/or management efficacy usually relate to small geographical area and therefore are unable to reflect variation that likely occurs across larger geographic areas. This characteristic of data along with variation in methods and specific questions examined make it difficult to discern the sources of variation in results summarized in this review. Careful experimental design, more extensive data collection, meta-analytics of past results, and other approaches could improve our understanding of the sources of variation in ECF results. In particular, care should be taken to control for endogenous management response to ECF risk and infection at the household level in order to uncover the causal relationships between the underlying drivers of ECF burden, management responses to it, and the incidence and distribution of ECF illness and economic consequence.

### 7.5. Scope of ECF impact on domestic animals

The adoption of ECF immunization for cattle may affect the productivity of other animals. Dantas-Torres and Otranto (72) report that parasites related to *T. parva* have also seen in cats, horses, and donkeys. The expenditure for preventing ECF also can influence the adoption of other animals vaccination due to limited resources. However, the literature has rarely looked at the spillover effects of the adoption of ECF immunization for cattle and other animals disease/production. This is a theoretical and practical knowledge gap.

### 7.6. The role of institutions in ECF vaccination distribution

Perry (49) provides a useful history of the development of the Muguga Cocktail. Several non-government organizations (NGOs) or environmental, social, and governance (ESG) associations are becoming important for African agriculture. NGOs have played a major role in providing animal health care. For example, if cattle are owned by cooperatives or unions, NGOs can provide systematic and personalized assistance. Through experience, they can make households better off by providing cattle healthcare services and consequently yield more milk. Sedlacek and Gaube (73) discuss that a regional association becomes a pivotal player in the regional development process by supporting cooperation between regional stakeholders. It provides information and news for a new disease or treatment method. Regional stakeholders can easily communicate for their business under the organization. Thus, it might be interesting and important to analyze the role of such association for ECF disease.

### 7.7. Improvement of supply and demand estimation in ECF Management

Randolph et al. (53) discussed above is the only paper identified in this review that examines the willingness to pay for two ECT prevention vaccines using conjoint analysis and contingent valuation. Because sufficient market data were not available to do market-based demand and willingness to pay analysis, they used survey-based non-market methods. While these methods are useful in the absence of market data, additional market-based analysis would help expand our understanding of the market demand for ITM and similar products.

Similarly, investments aiming to improve infrastructure for vaccine delivery are crucial. It would be interesting to examine how establishment of cold chains and better delivery network can increase vaccine accessibility and lower acquisition costs. In terms of ITM especially, estimates of the extent of the ITM market, the characteristics and drivers of demand and willingness to pay (WTP) can provide important insights to inform ITM supply decisions and policy around their implementation. For instance, if farmers are willing to bear a significant portion of the cost of ITM, then government can implement cost-sharing strategies like subsidies, grants and other financial services. In case of sufficient demand it could also create opportunities for private sector investments. Demand estimation studies can also highlight the need for education and awareness so that farmers are equipped to make informed decisions regarding ITM strategies. Additional clarity on the supply side of the ITM market would also be valuable, especially a clear understanding of the costs of production and delivery and barriers to access of ITM for important cattle regions.

### 7.8. Gender and other demographic drivers of management

There is little information on demographic and other drivers of adoption and willingness to pay (WTP) for ITM against ECF. As described in Section 4.2, Jumba et al. (54) finds differences in adoption by gender, age, education, credit access, and other factors. The cost of the vaccine and awareness were major constraints for women, whereas the bulk dosage availability in packages of 40 apparently hinders adoption of ITM among men. However, these findings are based on a small sample size. In addition, in smallholder settings, women and men often influence production decision through a household decision-making process rather than through separate decisions. Additional research examining gender perspectives, intrahousehold decision-making, and other factors can validate findings and delve deeper into gender-related aspects and targeted policies.

### 7.9. Indirect economic impacts on households, communities, and regional economies

A few papers have focused on the indirect impacts borne by households due to the impact of EFC on the household. Marsh et al. (41) looked at the causal relationship between indirect benefits of adoption of ECF vaccines and improvement in household welfare in the form of childhood education and food purchase. Further research could look at how the indirect benefits of vaccination are distributed through the household. Female educational attainment, maternal health, and early childhood development and nutrition are key areas of interest. Moreover there are no studies in economic literature related to ECF that look at the separability between the household production and consumption decisions. It is important to look at how policies could be targeted to minimize production risks imposed by ECF to ensure optimal allocation of resources within a household.

### 7.10. On-farm labor costs of ECF management

On-farm labor costs may be a relatively large component of the costs of ECF management, but little research has been carried out to examine the opportunity cost of ECF prevention and treatment associated with animal husbandry time spent. Members of the household have to put in time and energy to take care of sick cattle which could have been otherwise used for productive activities. For instance, adult members taking care of the sick cattle could engage in allied agricultural activities if the cattle were healthy. The additional income by engaging in allied agricultural activities is lost. Similarly young children taking care of sick cattle could use the time to study and enhance their education and knowledge if the cattle were healthy. Hence, in terms of estimating benefits incurred by households from adoption of ECF vaccine, one could also look at time use. It would be specifically interesting to examine if reduction in the incidence of ECF helps adults and children of the household to engage in other productive activities. In addition, further research could be done in exploring household behavioral changes because of reduction in the burden due to ECF. One could look at decision-making related to intra-household allocation food and other resources and if there are in changes that households undertake related to production. This can provide an insight on the changes in behavioral and consumption patterns of households.

### 7.11. Herd portfolio, risk avoidance, and productivity

Although there is a broad understanding that ECF morbidity and mortality risk varies by cattle breed and age (28), little or no quantitative analysis has focused on the marginal tradeoffs between herd productivity in terms of milk production and market value gain against the morbidity and mortality differences across breeds and herd portfolios. For instance, farmers may choose to avoid highly productive but highly ECF-susceptible dairy breeds because of the ECF-based risks of holding these breeds. More generally, ECF could be acting as a barrier to adoption of new technologies and breeds, thus missing potentially productive investment opportunities (22).

### 7.12. Disease burden and trade

Animals that are ill with an infectious disease, especially visibly ill, may be disallowed to enter the cattle market, and/or are quarantined (74, 75). Further, ECF symptoms of weight loss, reduced cattle condition, the possibility of persistence reduce the market value of cattle, and quarantine itself can affect livestock prices (76). While the literature on morbidity costs covered in this review in some cases use market data for estimation of economic impacts, there are several dimensions of ECF impact on marketability and market value that are worthy of research. For example, the relationship between vaccine uptake and commercial off-take is relatively poorly understood (38).

## 8. Conclusion

This paper presents a review of existing literature that focuses on the economic burden of East Coast Fever. ECF is a tick borne disease of cattle that imposes a major burden for small scale livestock holders in Africa. We start by presenting a simple conceptual framework to calculate the economic cost of the disease. Theoretically the economic costs due to ECF can be obtained by summing several cost elements that consist of production losses, additional non veterinary costs, prevention and disease control costs. Followed by the introduction of cost elements, we explain what each of the cost elements consist of and how they interact with one another. Based on empirical literature we present studies that have looked at losses due to ECF, at the micro level, in the form of mortality and morbidity, and at the macro level. We then look at types, costs and benefits of different types of management strategies to control ECF. There are two major control methods, namely spraying acaricides and vaccination via the infection and treatment method. The only treatment strategy is use of antibiotics to curb ECF. We also look at studies that have explored indirect costs that households may incur due to the presence of ECF.

In order to understand the trade off between the control and preventative strategies we look at studies that have calculated the ECF disease burden. In terms of empirical literature, studies have used partial budget analysis, regression techniques and comparisons of means to estimate the burden of ECF. Based on existing studies we observe that spraying acaricides is the most traditional preventative method. However this method is expensive and not efficient as it enables ticks to develop resistance against it. The more efficient control strategy is infection and treatment method, ITM, which provides long-term immunity to cattle. We also observe that livestock owners that adopt vaccination experience net gains as opposed to the non-vaccinated households that incur losses. Moreover, vaccinating livestock owners also save more post immunization as they reduce the application of acaricides.

Lastly we identify knowledge gaps at aggregate and household levels. At the macro level, there are several constraints that are exogenous to households like establishment for infrastructure for vaccine delivery, presence of NGOs for access to subsidised vaccines etc. Investments in such infratructure can bring down the cost of vaccines and consequently make it more accessible and affordable. At the micro level, the existing techniques used to estimate economic costs of ECF have several shortcomings. Hence other econometric techniques should be considered to get a more accurate understanding of the same. Understanding the causal impact of animal diseases on welfare will be helpful for policy makers. Lastly research should be done on indirect costs caused by the presence of ECF. The downstream effects due to economic costs of ECF can be experienced by members of the household, particularly women and children. Research in this area requires attention it can provide insights when designing policies and interventions directed at decreasing poverty, improving food security, and consequently improving health.

## Author contributions

AS and JH carried out the literature search and were primarily responsible for the development of the draft text and its substantive content. SM, JO, and JY provided guidance on content and editorial review and revision. All authors contributed to the article and approved the submitted version.

## Footnotes

^1^The broader agenda is being pursued with support from the USAID Feed the Future Global Hunger and Food Security Initiative as noted in the acknowledgments.

^2^Various species of Theileria cause various illnesses in livestock and wildlife across the world. *T. annulata* is responsible for tropical theileriosis, one of the most significant of these (18). Tropical theileriosis has a wide distribution, including North Africa, southern Europe, the Middle East, central Asia, India, and China.

^3^Herd immunity is a special case of stability in which transmission and spread is very limited or approaches zero.

^4^2.9 million cattle were assumed to be immunised over a period of 30 years, 100,000 cattle per annum.

^5^All monetary values have been converted to 2023 US dollars (USD).

^6^The cattle population at risk (*p* in Equation 2) to estimate the total loss was reported to be in the range of 242–7,489.
